# Successful use of non-contrast dual energy computed tomography in patients with differentiated thyroid cancer

**DOI:** 10.3389/fendo.2026.1742341

**Published:** 2026-03-19

**Authors:** Adam Daniel Durma, Marek Saracyn, Arkadiusz Zegadło, Grzegorz Kamiński

**Affiliations:** 1 Department of Endocrinology and Radioisotope Therapy, Military Institute of Medicine – National Research Institute, Warsaw, Poland; 2 Department of Preclinical Sciences, Faculty of Medicine, University of Warsaw, Warsaw, Poland; 3 Department of Radiology, Military Institute of Medicine – National Research Institute, Warsaw, Poland

**Keywords:** non-contrast, DECT, dual energy computed tomography, DTC, differentiated thyroid cancer

## Abstract

**Background:**

Differentiated thyroid cancer (DTC) is the most commonly diagnosed endocrine cancer. Diagnosis of DTC metastases is possible with the use of ultrasound, RAI scintigraphy, [^18^F]FDG PET/CT, or contrast-enhanced CT; however, the use of iodine contrast factors (ICF) delays potential RAI treatment. Dual energy computed tomography (DECT) is a variant of computed tomography that enables the detection and calculation of iodine concentration in tissues. The study aimed to evaluate the potential use of non-contrast DECT in diagnosing DTC metastases.

**Materials and methods:**

This prospective study enrolled 37 patients who had undergone thyroidectomy for DTC and were found to have lesions suspected of being metastatic. DECT was performed at least six months after the last administration of RAI or ICF. Group differences were analyzed using statistical tests, including the Student’s t-test and the Mann-Whitney U test. Receiver operating characteristic (ROC) curves were utilized to assess the sensitivity and specificity of selected parameters.

**Results:**

In 31 of 37 patients, non-contrast DECT confirmed increased accumulation of endogenous iodine. Per patient, DECT sensitivity was 93.5%, specificity was 100%, positive predictive value (PPV) was 100%, and negative predictive value (NPV) was 71.4%. Statistically higher values of iodine concentration (IC), effective atomic number (Z_eff_), and Hounsfield Unit (HU) were observed for DTC metastases compared to normal lymph nodes. The area under the curve (AUC) for endogenous iodine concentration was 0.992, and a threshold of endogenous IC >250 µg/cm3 provided a sensitivity of 96.6% and a specificity of 87.0% for detecting DTC metastases.

**Conclusions:**

Non-contrast DECT is useful in the diagnosis of DTC metastases, demonstrating high sensitivity and specificity. A key advantage is the lack of necessity for ICF, which prevents delay in potential radioiodine therapy and is safer for patients with an allergy to such factors.

## Background

Differentiated thyroid cancer (DTC) is the most commonly diagnosed endocrine cancer worldwide (1). Treatment involves thyroidectomy with lymphadenectomy and subsequent radioiodine (RAI) treatment (RIT) (2). RAI is used due to its ability to accumulate in thyroid cancer metastases similarly to normal thyrocytes (3). Diagnosis of DTC metastases is possible with the use of RAI scintigraphy, [^18^F]FDG PET/CT, and contrast-enhanced CT; however, the use of iodine contrast factors (ICF) delays potential RIT (4–6).

Dual-energy computed tomography (DECT) is an advanced imaging technique used for both qualitative and quantitative analysis of tissues and materials. It leverages specialized features of certain computed tomography (CT) scanners and their software to differentiate tissue properties. Introduced by Flohr in 2006 (7), DECT differs from standard CT in both imaging protocols and post-scan software analysis, enabling assessment based on the atomic numbers of tissues. In conventional CT, tissues are evaluated based on their elemental composition, with results displayed as pixel values reflecting mass density (8, 9). In contrast, DECT utilizes two X-ray spectra at different energy levels to capture images, enabling enhanced material differentiation. Although protocols vary by CT manufacturer, the core principle remains consistent (10): energy levels ranging from 40 keV to 140 keV are alternately transmitted through the object to the CT detectors, and specialized software processes this data for image analysis and presentation. One example of DECT technology is Gemstone Spectral Imaging (GSI) by General Electric (GE) (11–13). GSI employs dual-energy levels to analyze the attenuation properties of materials. During a single scan, data is collected from detectors at two distinct X-ray levels, which is then processed by image reconstruction software to enhance tissue differentiation and detail. This technique allows for precise material characterization by targeting specific regions of interest (ROIs) and can even highlight the “atomic density” of tissues (10). The most common elements in the human body have low atomic numbers, including hydrogen, carbon, nitrogen, and oxygen. Since water (H_2_O) constitutes a large portion of the body’s composition, DECT is particularly effective in distinguishing tissues with varying atomic compositions, especially those containing elements with higher atomic numbers like iodine (14). Currently, DECT is widely used with iodine-based contrast agents, given iodine’s high atomic number and its frequent application in diagnostic imaging (15). Recent studies have demonstrated DECT’s expanding utility in fields such as orthopedics, oncology, and vascular diagnostics (16–21). These studies utilize iodine contrast agents to highlight targeted tissues and assess additional parameters, including iodine concentration (IC), normalized iodine concentration (NIC), spectral Hounsfield unit curve slope (λHU), and effective atomic number (Z_eff_).

Certain tissues, such as the thyroid gland, have distinctive elemental compositions that further enhance DECT’s imaging capabilities. The thyroid relies heavily on iodine for triiodothyronine (T3) and thyroxine (T4) production, absorbing it from the gastrointestinal tract or intravenously administered iodide. Iodide is transported into thyrocytes via a sodium-iodide symporter, which facilitates the entry of two sodium and one iodide ions into the cell (22, 23). Inside the cell, iodide undergoes oxidation by thyroid peroxidase (TPO) and integrates into thyroglobulin to store thyroid hormones. The high iodine concentration in thyroid tissues enables their easy detection with DECT, as iodine-rich areas appear prominently on iodine-water maps.

In a 2012 study, Li et al. used DECT to examine 97 surgically excised thyroid nodule specimens prior to pathological analysis. They compared nodular goiters, follicular adenomas, and papillary carcinomas, observing a significant difference in iodine concentration between benign and malignant nodules (p < 0.001) as well as in other DECT-derived parameters (24). This study suggested that iodine concentration can vary notably between benign and malignant tissues, even in studies conducted without ICFs. Although studies have demonstrated DECT’s utility in evaluating thyroid tissue, its application has largely relied on the administration of ICFs, which are selectively absorbed by thyroid tissue (25–27). From a pathophysiological perspective, thyroid-related tissues, including metastases of differentiated thyroid cancer (DTC), should theoretically be capable of accumulating “endogenous” iodine, assuming the sodium-iodide symporter is functioning properly. However, in cases of advanced DTC, the use of ICFs may delay subsequent RIT. Additionally, ICFs can sometimes trigger allergic reactions, including anaphylactic shock, and are contraindicated for patients with advanced chronic kidney disease (6, 28–30). So, the method that differentiates metastatic and reactive lymph nodes in patients with DTC after thyroidectomy without using ICFs reduces the number of invasive procedures as well as the frequency of follow-up visits.

Considering the aforementioned, we hypothesized that DECT might be suitable for confirmation of suspected DTC metastases, even without the use of ICFs. To explore this idea, we initially applied non-contrast DECT to diagnose and monitor two patients with DTC—one with papillary thyroid cancer (PTC) and the other with follicular thyroid cancer (FTC) (31). The results ultimately encouraged us to conduct a prospective study assessing the potential utility of non-contrast DECT in patients with DTC.

## Methods

### Study aim

The aim of this study was to evaluate the potential use of non-contrast DECT in the diagnosis of DTC metastases.

### Study group

The study group enrolled 37 patients of the highest-reference center prospectively qualified between 2022 and 2024, with a diagnosis of DTC. Patients were at least 6 months after thyroidectomy and RIT, and during follow-up suspicious lymph nodes or lung nodules were found. Lymph nodes were found by ultrasound (USG), and one lung nodule was found by [^18^F]FDG PET/CT. Patients underwent non-contrast DECT at least 6 months after any iodine intake (RIT/ICF/iodine supplements).

Patients were hospitalized in accordance with a local protocol of DTC follow-up. Day one, measurement of thyroglobulin (Tg) and anti-thyroglobulin titer (aTg) were performed. Next, on day one and day two, 0.9mg of recombinant human thyroid-stimulating hormone (rhTSH; Thyrogen^®^) were given intramuscularly. On day two, DECT was also additionally performed. Concentration of stimulated thyroglobulin (sTg) was measured on day 3. On day 6, patients underwent RAI scintigraphy (2.3mCi of 131I). If there was a suspicion of non-iodine-avid metastases, determined by sTg > 5 ng/dL, [^18^F]FDG PET/CT was also referred.

The diagnostic process led to surgery in 31 patients, where histological results were also obtained. As during DECT studies, no ICFs were used, in this paper, we defined endogenous iodine concentration (eIC), which in technical aspect is iodine concentration (IC), but with no use of contrast enhancement.

### Computed tomography protocol

Tests were performed using a Discovery CT 750 HD kVp fast-switching single-source spectral CT (SpCT) scanner (GE Healthcare, WI, USA). The studies were transferred from the scanner to a commercially available Advantage Workstation server 4.7 (GE Healthcare) for processing, following the Gemstone Spectral Imaging (GSI) General protocol in GE Healthcare Workstation 4.7 software.

Regions of interest (ROIs) were conjointly selected by two of the authors. Initially, thyroid USG was performed to identify suspicious lymph nodes (the group of lymph nodes (I-VI), their size, and distance from other structures were assessed). Subsequently, after performing a DECT scan, the previously obtained USG results were correlated with the CT scan, identifying previously observed suspicious lymph nodes and their surroundings. A significant difference in endogenous iodine concentration compared to the surrounding tissues suggested a suspected metastasis. Since the Ethics Committee did not allow the use of healthy patients as a control group, non-suspicious lymph nodes were identified in the CT scans of the patients as “control nodes = non-suspicious” (e.g., submandibular, posterior cervical, submental).

The results of iodine concentration (IC; µg/cm³) were automatically generated and displayed as the mean concentrations of endogenous iodine in the ROI corresponding to the measurement site. ROI was covering all the lesions and was manually measured by a radiologist and an endocrinologist. Moreover, the water concentration (H_2_O; mg/cm3), Hounsfield Unit (HU) values, and effective atomic number (Z_eff_) were additionally measured. The tests were color-coded using Inverse Gray and GE Colormap to enhance positional assessment and contrast, a feature available in the GSI General software on the Advantage Workstation (AW).

### Statistical analysis

The statistical analysis was performed using IBM SPSS (version 26). To verify whether the results met the rules of normal distribution, the Shapiro-Wilk test was conducted. Results with normal distribution were presented as means (M) and standard deviations (SD), and those of non-normal distribution as medians (Med.) and interquartile ranges (IQR). Differences between groups were analyzed using appropriate tests, such as t-Student tests and the U Mann-Whitney test. The Levene test was used to assess the equality of variances in the analyzed groups. The Pearson test was used for correlation analysis. ROC curves were used for the assessment of the sensitivity and specificity of the selected parameters. Results were considered statistically significant for a value of α less than 0.05.

## Results

The study group included 23 (62.2%) females and 14 (37.8%) males. Mean age was 49.5 ± 17.8. Thirty-four patients had PTC, two had FTC, and one had poorly differentiated thyroid cancer. The initial TNM details of the study group are presented in **Table 1**. The mean summary radioiodine activity that was given to patients prior to study enrollment was 194.1 ± 108.5 mCi. DECT studies provided a mean radiation dose of 1070.0 ± 356.0 mGycm, which was equal to 6.313 ± 2.100 mSv.

**Table 1:** Initial T(m)NM details of the study group.

Staging	Parameters	Number of patients
T	1a	15
	1b	7
	2	2
	3	12
	4	1
m	Yes	8
	No	29
N	0	10
	1	27
M	0	36
	1	1

T, tumor; m, multifocal; N, nodule; M, metastases.

In 83.8% (31/37) of patients, DECT confirmed increased accumulation of iodine in suspicious lymph nodes. In this group, RAI scintigraphy showed accumulation only in 16.7% (6/36) cases (in one patient, scintigraphy was waived due to visible trachea and blood vessel infiltration).

In the subgroup with “negative” DECT (n=6), five patients had excellent response to treatment and no biochemical or imaging presence of DTC [confirmed by measurement of: Tg, aTg, fine needle aspiration (FNA) washout Tg, fine needle aspiration cytology (FNAC), RAI scintigraphy and follow-up USG after 3–6 months] - were considered as true negatives.

In 1 patient, DTC metastases visible in USG (but not visible in DECT) were confirmed by lymph node FNAC, and subsequently by surgery - the study was considered a false negative.

**Figure 1:**
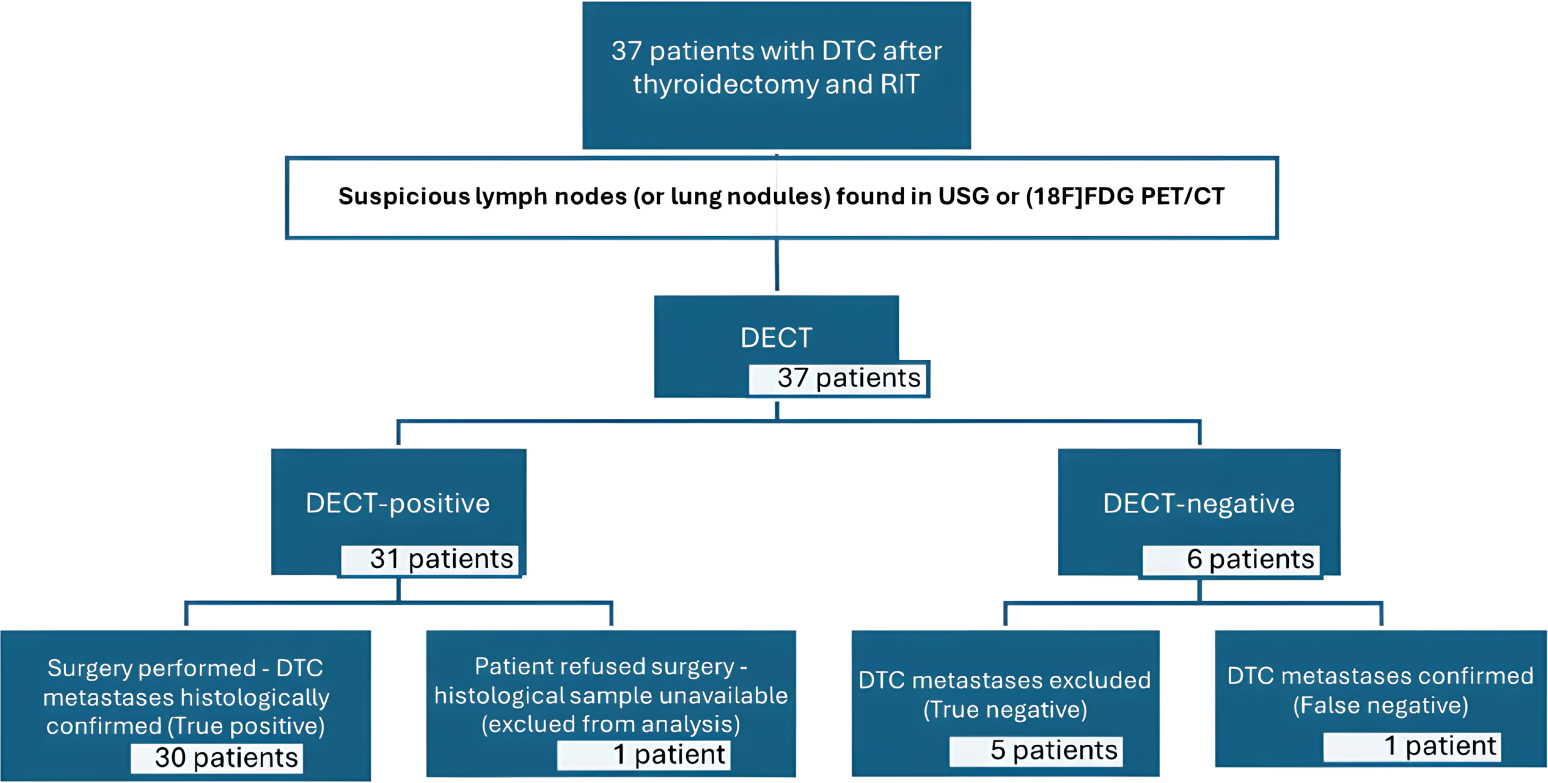
Diagnostic chart describing study group. DTC, differentiated thyroid cancer; RIT, radioiodine treatment; DECT, dual energy computed tomography.

One patient with positive DECT (1/31) refused the surgery and decided to remain under clinical observation (Tg was 0.06 ng/dL, sTg was 0.17ng/dL; DECT eIC 520 µg/cm3). This patient was excluded from the statistical analysis and calculations. Considering the aforementioned DECT specificity, sensitivity, positive predictive value (PPV), negative predictive value (NPV), and accuracy were calculated based on a group of 36 patients with confirmed disease status. The results per patient were: Specificity = 93.5%, sensitivity= 100%, positive predictive value (PPV)= 100%, negative predictive value (NPV = 71.4%), and accuracy 94.4%. The diagnostic chart is presented in **Figure 1**.

**Figure 2:**
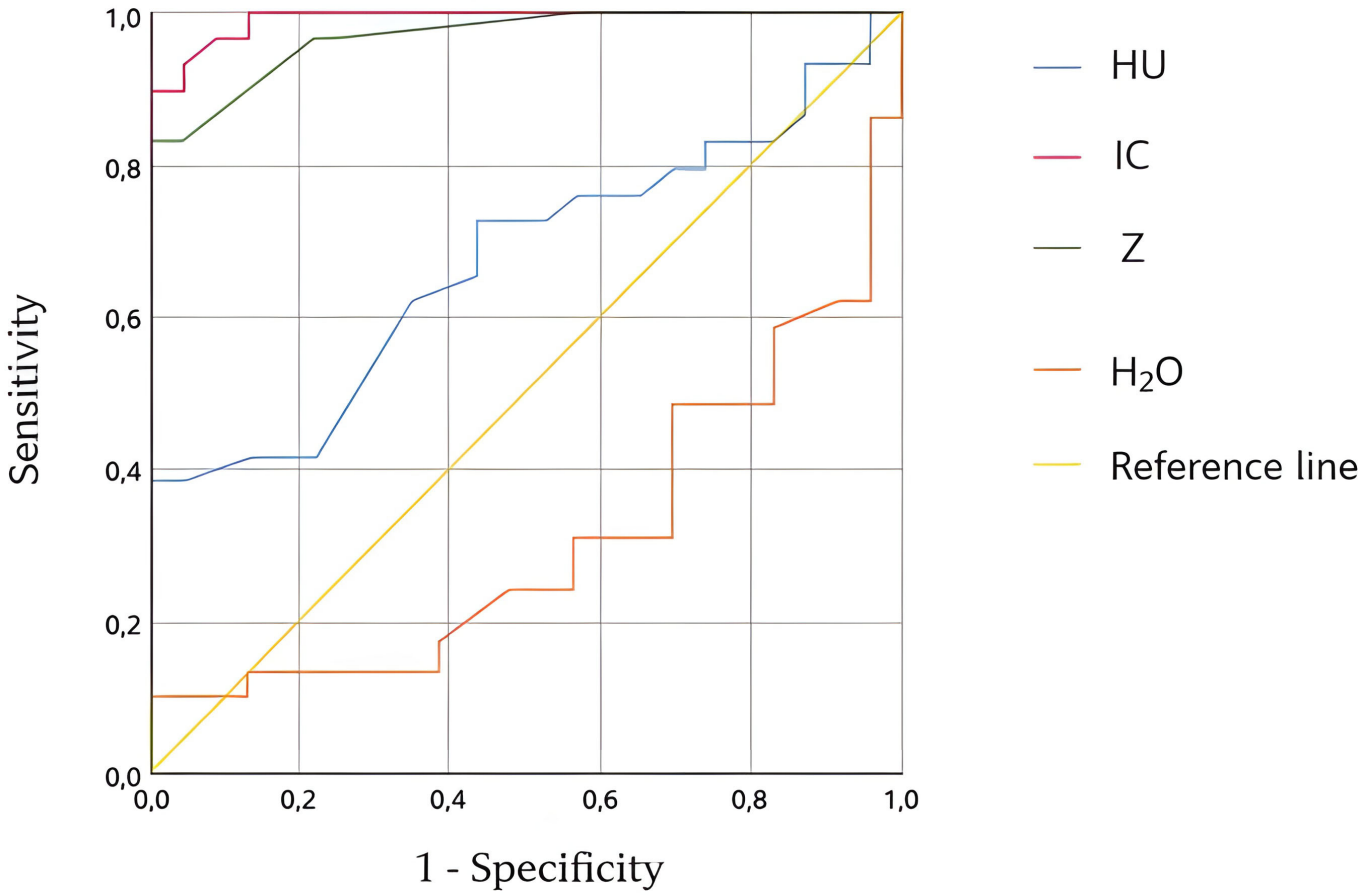
ROC curves for IC, Z, HU and H_2_O. ROC, receiver operating characteristic; IC, endogenous iodine concentration; Z-effective atomic number; HU, Hounsfield units; H_2_O, water concentration.

For the final *post-hoc* analysis, we have selected 80 lymph nodes or lung lesions –50 that were considered as suspicious ones, and 30 normal ones that were considered as control (“normal”) ones - the detailed description of their parameters is presented in **Table 2**. DECT parameters showed statistically significant differences in HU, eIC, H2O, and Z_eff_ depending on lesion type. Nevertheless, the most important and accurate parameters appeared to be IC, Z_eff_, and HU. The detailed parameters are presented in **Figure 2**; **Tables 3**, **4**.

**Table 2:** Comparison of data regarding suspicious (DTC) lymph nodes/lung foci and normal lymph nodes.

Parameter	Unit	Suspicious lymph nodes/foci (n=50)		Normal lymph nodes (n=30)		Δ	p
		M	SD	M	SD		
HU	NA	40.2	21.2	30.2	20.3	-10.0	0.041
eIC	µg/cm³	938.5	721.3	-16.6	344.6	-955.1	<0.001
H_2_0	mg/cm³	1021.6	25.0	1031.6	12.6	10	0.021
Z_eff_	NA	8.31	0.56	7.47	0.36	-0.84	<0.001

HU, Hounsfield units; IC, endogenous iodine concentration; H_2_O, water-water parameter; Z_eff_, effective atomic number; M, mean; SD, standard deviation; Δ, difference; p, p-value; NA, not applicable.

**Table 3:** Area under curve and its parameters for HU, IC, Z and H_2_O.

Parameter	AUC	p	CI 95%	
HU	0.672	0.034	0.526	0.819
eIC	0.992	<0.001	0.977	1.000
Z_eff_	0.968	<0.001	0.928	1.000
H_2_O	0.309	0.019	0.163	0.454

HU, Hounsfield units; eIC, endogenous iodine concentration; H_2_O, water-water parameter; Z_eff_, effective atomic number; AUC, area under curve; CI, confidence interval; p, p-value.

**Table 4:** Sensitivity and specificity given by ROC curve for, eIC, Z_eff_, HU and H_2_O.

Parameter	Value	Sensitivity	Specificity
HU	17.0	82.8	26.1
eIC	250.0	96.6	87.0
Z_eff_	7.68	96.6	72.9
H_2_0	1019.0	62.1	8.7

HU, Hounsfield units; eIC, endogenous iodine concentration; H_2_O, water-water parameter; Z_eff_, effective atomic number.

In the subgroup with histologically confirmed DTC metastases, median (with IQR) Tg, sTg, and aTg, were 0.65 (2.88) ng/dL, 5.0 (30.4) ng/dL, and 16.0 (6.0) ng/dL, respectively.

DECT H_2_O parameter in the metastatic lesions correlated negatively with IC (p<0.001; R = 0.550), while Z_eff_ and aTg correlated positively with IC (p < 0.001; R = 0.588; and p=0.032; R = 0.301 respectively).

## Discussion

Our study demonstrated the significant utility of the DECT method in diagnosing DTC metastases. Notably, in this small study group, the method exhibited very high sensitivity and specificity - 93.5% and 100%, respectively. Due to the study’s innovative approach, which involved the absence of ICFs, there were no established cutoff values for comparison. While histological examination after surgical excision is a gold standard in confirming metastasis, a partial confirmation that a suspicious lesion could be a DTC metastasis could be achieved through verification with other available methods, such as FNAC or [^18^F]FDG PET/CT. Moreover, biochemical monitoring of parameters like Tg or aTg could also bring some insight into the potential stage of the disease. The use of contrast-enhanced computed tomography delays the possibility of RIT for 3–6 months, which is why DECT was chosen as an alternative method of DTC metastases diagnostics.

The analysis of lymph nodes allowed for an estimation of the potential sensitivity and specificity of the DECT method, which exceeded 90% at an endogenous iodine concentration >250 µg/cm³—a promising result that supports the method’s potential future use. The study also highlighted that patients for whom iodine-based contrast-enhanced imaging is either unfeasible or contraindicated (e.g., due to contrast allergies or renal failure) may benefit from DECT. Importantly, the examination does not delay any potential adjuvant RIT.

The primary drawback of this study appears to be the diagnostic process itself, which requires close collaboration between a clinician/sonographer capable of identifying a potentially suspicious lesion and an experienced radiologist skilled in CT analysis. In a contrast-free examination, the radiologist must accurately confirm the previously detected lesion’s location and perform a numerical analysis of its parameters by placing a region of interest (ROI). While this level of collaboration may pose a challenge, it does not diminish the value of the method—particularly for confirming lesion characteristics when other available diagnostic approaches fail or yield inconclusive results. Additionally, the method holds particular relevance for specific patient groups, such as those previously mentioned, for whom conventional contrast-enhanced imaging is not feasible.

The one patient with a false negative DECT study had PTC, and found in follow-up a 9x8x6mm lymph node in the IV sector of neck lymph nodes, with mixed vascularity. The possible reason for a false negative study might be the size of the lymph node itself, or failure of the radiologist to properly locate the lesion.

Yao et al., in their retrospective study on 55 patients, aimed to determine whether GSI contrast-enhanced CT with virtual non-contrast (VNC) images could reliably aid in characterizing thyroid lesions (distinguishing thyroid papillary carcinoma from nodular goiter) (32). Papillary carcinoma showed significantly lower absolute attenuation between VNC and true non-contrast (TNC) than nodular goiter (7.86 ± 6.74 HU vs. 13.43 ± 10.53 HU, p=0.026). Similar results were observed for IC (3145 ± 851 µg/cm3 vs. 3727 ± 1034 µg/cm3, *p* = 0.016). The iodine density showed higher diagnostic performance (AUC = 0.727), accuracy (0.773 vs. 0.667), sensitivity (0.750 vs. 0.708), and specificity (0.786 vs. 0.643) than the absolute attenuation between TNC and VNC images (AUC = 0.683). In another study Li et al., in a retrospective study including 150 patients with thyroid nodules underwent preoperative DECT and Thyroid Imaging Report and Data System (TIRADS) classification (33). Finally, 96 patients had malignant tumors while 54 had benign ones. For thyroid nodules the IC and NIC cut-off values were IC_a_ (2.835 mg/mL), NIC_1a_ (0.690), and their corresponding area under the curve (AUC) were 0.940, 0.954 respectively. For lymph nodes thresholds were IC_a_ (1.715 mg/mL) and NIC_2a_ (0.155), and their corresponding AUC were 0.717, 0.720 respectively.

In another study using contrast DECT Ha et al. retrospectively enrolled 63 patients to assess the utility of DECT in differentiating pulmonary metastasis and benign lung nodules in thyroid cancer patients (34). If nodules showed increased RAI uptake on SPECT/CT or increased size in follow-up CT, they were considered metastatic. The DECT parameters of the metastatic nodules were significantly higher than those of the benign nodules (IC, 5.61 ± 2.02 mg/mL vs. 1.61 ± 0.98 mg/mL; NIC, 0.60 ± 0.20 vs. 0.16 ± 0.11; NIC using pulmonary artery [NICPA], 0.60 ± 0.44 vs. 0.15 ± 0.11; and Zeff, 10.0 ± 0.94 vs. 8.79 ± 0.75; all p < 0.001). The cutoff values for IC, NIC, NICPA, and Zeff for diagnosing metastases were 3.10, 0.29, 0.28, and 9.34, respectively. Zou et al. retrospectively included 406 patients in whom DECT parameters also proved to be independent indicators of PTC lymph node metastases (35). Yang et al. used DECT for differentiating malignant cervical lymphadenopathy caused by DTC, salivary gland carcinoma, squamous cell carcinoma and lymphoma in 92 patients (36). Results showed that IC and HU slope of DTC subgroup were significantly higher than those for other neoplasms (p < 0.001). In contrast-enhanced images mean IC for DTC lymph nodes was 3985 ± 1457 µg/cm3. Zhao et al. in 2024 performed a retrospective analysis of 140 patients with PTC and negative diagnosis of cervical central lymph node metastases by preoperative evaluation (37). Patients underwent preoperatively contrast-enhanced DECT and results showed significant differences in DECT parameters for lesions that appeared to be metastatic in post-operation histological examination, especially in the arterial phase (IC, NIC, Zeff, HU slope). Alternberd et al. in the study including 70 patients in whom 156 pulmonary lesions were assessed showed significant differences in DECT parameters allowing for the detection of different types of metastases (e.g. melanomas, thyroid, or colorectal cancers) (38).

The study by Wu et al. included 35 patients with histologically proven PTC that underwent DECT studies prior to thyroidectomy and lymphadenectomy (39). In total, they assessed 206 lymph nodes (80 metastatic and 126 benign). The best single DECT parameter for DTC metastases confirmation was venous phase NIC, with a sensitivity of 62.5% and a specificity of 85.7%. On the other hand, He et al. in their study showed that the best quantitative parameter for detection of PTC lymph node metastases might be arterial-phase NIC (40). Jin et al. in a retrospective study analyzed 293 lymph nodes from 78 patients with papillary thyroid cancer (PTC) to develop a deep learning model (41). The results showed that the model integrating both CT images and iodine maps of the arterial and venous phases showed good performance in predicting the presence of PTC metastases. DECT parameters also appeared to be useful in differentiating PTC metastases in patients with Hashimoto’s thyroiditis (42).

To the best of our knowledge, our study was the first prospective one utilizing non-contrast DECT in the diagnosis of DTC metastases. As proven before, the DECT was a useful tool in assessment of this group of patients, however, our study proved the usefulness of the method without the need for ICF administration. The values of DECT parameters in our study were lower than in other studies probably because of the lack of ICF administration.

Nevertheless, the obtained results provided significant additional information, allowing for the detection of suspicious lymph nodes and metastatic foci. This knowledge made it possible to plan potential surgical procedures more precisely, as well as to reduce patient stress related to the presence of suspicious nodules or lung foci. Due to the non-invasive nature of the test, the acceptable radiation dose, and the lack of potential complications, we recommend this method for everyday clinical practice, especially in uncertain or borderline cases. Despite the relatively small study group, the results are encouraging for further evaluation of the method and continuation of the study in different populations, particularly those in countries where salt iodization is not common.

The main limitations of the study are the small sample size and the single-center experience, which may affect the precision of sensitivity and specificity estimates. In addition, the proposed iodine concentration threshold (>250 µg/cm³) was arbitrarily adopted and requires validation in further studies. The results should be interpreted with caution, as the high sensitivity and specificity observed may be lower in larger patient cohorts. Nevertheless, the method remains promising for the assessment of a specific group of patients with DTC. Another limitation of non-contrast DECT is its strong dependence on expert radiologists, as well as the need for specialized equipment and software for image analysis.

## Conclusions

Non-contrast DECT appeared to be a useful tool in the diagnosis of DTC metastases, demonstrating high sensitivity and specificity in identifying suspicious lymph nodes and pulmonary lesions.

A key advantage of the method is the lack of ICF administration, which prevents delays of potential radioiodine therapy, and is safer for patients with an allergy to ICFs.

Further, multicenter studies are required to determine the usefulness of the method.

## Data Availability

The raw data supporting the conclusions of this article will be made available by the authors, without undue reservation.

## References

[1] Li M Dal Maso L Pizzato M Vaccarella S . Evolving epidemiological patterns of thyroid cancer and estimates of overdiagnosis in 2013–17 in 63 countries worldwide: a population-based study. Lancet Diabetes Endocrinol. (2024) 12:824–36. doi: 10.1016/S2213-8587(24)00223-7, PMID: 39389067

[2] Boucai L Zafereo M Cabanillas ME . Thyroid cancer: a review. JAMA. (2024) 331:425–35. doi: 10.1001/jama.2023.26348, PMID: 38319329

[3] Volpe F Nappi C Zampella E . Current advances in radioactive iodine-refractory differentiated thyroid cancer. Curr Oncol. (2024) 31:3870–84. doi: 10.3390/curroncol31070286, PMID: 39057158 PMC11276085

[4] Krajewska J Chmielik E Dedecjus M Jarząb B Hubalewska-Dydejczyk A Karbownik-Lewińska M . Diagnosis and treatment of thyroid cancer in adult patients - Recommendations of Polish Scientific Societies and the National Oncological Strategy. Update of the 2022 Update. Endokrynol. Pol. (2022) 73(5):799–802. doi: 10.5603/EP.a2022.0087, PMID: 37067538

[5] Jarząb B Dedecjus M Lewiński A . Diagnosis and treatment of thyroid cancer in adult patients – recommendations of Polish Scientific Societies and the National Oncological Strategy. 2022 update. Endokrynol. Pol. (2022) 73:173–300. doi: 10.5603/EP.a2022.0087, PMID: 35593680

[6] Sohn S.Y. Choi J.H. Kim N.K. Joung J.Y. Cho Y.Y. Park S.M. . The impact of iodinated contrast agent administered during preoperative computed tomography scan on body iodine pool in patients with differentiated thyroid cancer preparing for radioactive iodine treatment. Thyroid. (2014) 24(5):872–7. doi: 10.1089/thy.2013.0238, PMID: 24295076

[7] Flohr T.G. McCollough C.H. Bruder H. Petersilka M. Gruber K. Süss C. . First performance evaluation of a dual-source CT (DSCT) system. Eur. Radiol. (2006) 16(2):256–68. doi: 10.1007/s00330-005-2919-2, PMID: 16341833

[8] Seeram E. . Computed tomography: a technical review. Radiol. Technol. (2018) 89:279CT–302CT. 29298954

[9] So A Nicolaou S . Spectral computed tomography: fundamental principles and recent developments. Korean J Radiol. (2021) 22:86–96. doi: 10.3348/kjr.2020.0144, PMID: 32932564 PMC7772378

[10] McCollough CH Leng S Yu L Fletcher JG . Dual-, multi-energy CT: principles, technical approaches, and clinical applications. Radiology. (2015) 276:637–53. doi: 10.1148/radiol.2015142631, PMID: 26302388 PMC4557396

[11] Forghani R De Man B Gupta R . Dual-energy computed tomography: physical principles, approaches, usage, and implementation: part 1. Neuroimaging Clin N Am. (2017) 27:371–84. doi: 10.1016/j.nic.2017.03.002, PMID: 28711199

[12] Cheng H.W. Geng J.H. Tan Z.W. Wu W.Z. Hu X.L. Gong J.F. . The Application Value of Gemstone Spectral Imaging (GSI) Combined with an 80 mm Wide-body Detector in Head-neck CTA. Curr. Med. Imaging. (2024) 20:e15734056186139. doi: 10.2174/0115734056186139231101063906, PMID: 37957875

[13] Magee D. Jeewa F. Chau M.V.D. Loh P.L. Ballesta Martinez B. Saluja M. . Demonstrating the Efficacy of Dual Energy Computer Tomography with Gemstone Spectral Imaging Software to Determine Mixed and Single Composition ex vivo Urolithiasis. Res. Rep. Urol. (2024) 16:215–24. doi: 10.2147/RRU.S473167, PMID: 39345800 PMC11439343

[14] García-Figueiras R. Oleaga L. Broncano J. Tardáguila G. Fernández-Pérez G. Vañó E. . What to Expect (and What Not) from Dual-Energy CT Imaging Now and in the Future? J. Imaging. (2024) 10(7):154. doi: 10.3390/jimaging10070154, PMID: 39057725 PMC11278514

[15] Forghani R De Man B Gupta R . Dual-energy computed tomography: physical principles, approaches, usage, and implementation: part 2. Neuroimaging Clin N Am. (2017) 27:385–400. doi: 10.1016/j.nic.2017.03.003, PMID: 28711200

[16] Sauerbeck J Adam G Meyer M . Spectral CT in oncology. Rofo. (2023) 195:21–9. doi: 10.1055/a-1902-9949, PMID: 36167316

[17] Forghani R . Advanced dual-energy CT for head and neck cancer imaging. Expert Rev Anticancer Ther. (2015) 15:1489–501. doi: 10.1586/14737140.2015.1108193, PMID: 26535613

[18] Demirler Simsir B Danse E Coche E . Benefit of dual-layer spectral CT in emergency imaging. Clin Radiol. (2020) 75:886–902. doi: 10.1016/j.crad.2020.06.012, PMID: 32690242

[19] Chen M. Jiang Y. Zhou X. Wu D. Xie Q. . Dual-Energy Computed Tomography in Detecting and Predicting Lymph Node Metastasis in Malignant Tumor Patients: A Comprehensive Review. Diagnostics. (2024) 14(4):377. doi: 10.3390/diagnostics14040377, PMID: 38396416 PMC10888055

[20] Machida H. Tanaka I. Fukui R. Shen Y. Ishikawa T. Tate E. . Dual-Energy Spectral CT: Various Clinical Vascular Applications. Radiographics. (2016) 36(4):1215–32. doi: 10.1148/rg.2016150185, PMID: 27399244

[21] Suntharalingam S. Stenzel E. Wetter A. Guberina N. Umutlu L. Schlosser T. . Third generation dual-energy CT with 80/150 Sn kV for head and neck tumor imaging. Acta Radiol. (2019) 60(5):586–92. doi: 10.1177/0284185118788896, PMID: 30089396

[22] Boron WF Boulpaep EL . Medical Physiology: A Cellular and Molecular Approach. 1st ed. Philadelphia: Elsevier/Saunders (2003). p. 1300.

[23] Melmed S Polonsky KS Larsen PR Kronenberg HM . Williams Textbook of Endocrinology. 12th ed. Philadelphia: Saunders (2011). p. 331.

[24] Li M. Zheng X. Li J. Yang Y. Lu C. Xu H. . Dual-energy computed tomography imaging of thyroid nodule specimens: comparison with pathologic findings. Invest. Radiol. (2012) 47(1):58–64. doi: 10.1097/RLI.0b013e318229fef3, PMID: 21788907

[25] Zhou Y. Xu Y.K. Geng D. Wang J.W. Chen X.B. Si Y. . Added value of arterial enhancement fraction derived from dual-energy computed tomography for preoperative diagnosis of cervical lymph node metastasis in papillary thyroid cancer: initial results. Eur. Radiol. (2024) 34(2):1292–301. doi: 10.1007/s00330-023-10109-0, PMID: 37589903

[26] Chen W. Lin G. Cheng F. Kong C. Li X. Zhong Y. . Development and Validation of a Dual-Energy CT-Based Model for Predicting the Number of Central Lymph Node Metastases in Clinically Node-Negative Papillary Thyroid Carcinoma. Acad. Radiol. (2024) 31(1):142–56. doi: 10.1016/j.acra.2023.04.038, PMID: 37280128

[27] Li L. Cheng S.N. Zhao Y.F. Wang X.Y. Luo D.H. Wang Y. . Diagnostic accuracy of single-source dual-energy computed tomography and ultrasonography for detection of lateral cervical lymph node metastases of papillary thyroid carcinoma. J. Thorac. Dis. (2019) 11(12):5032–41. doi: 10.21037/jtd.2019.12.45, PMID: 32030219 PMC6988027

[28] Fang S. Han S. Zhang P. Zhao C. . Effects of Iodinated Contrast-enhanced CT on Urinary Iodine Levels in Postoperative Patients with Differentiated Thyroid Cancer. Curr. Med. Imaging. (2024) 20:e15734056287560. doi: 10.2174/0115734056287560240117092339, PMID: 39185655

[29] Davenport M.S. Perazella M.A. Yee J. Dillman J.R. Fine D. McDonald R.J. . Use of Intravenous Iodinated Contrast Media in Patients with Kidney Disease: Consensus Statements from the American College of Radiology and the National Kidney Foundation. Radiology. (2020) 294(3):660–8. doi: 10.1148/radiol.2019192094, PMID: 31961246

[30] Fukushima Y. Taketomi-Takahashi A. Suto T. Hirasawa H. Tsushima Y. . Clinical features and risk factors of iodinated contrast media (ICM)-induced anaphylaxis. Eur. J. Radiol. (2023) 164:110880. doi: 10.1016/j.ejrad.2023.110880, PMID: 37187078

[31] Durma AD Saracyn M Zegadło A Kamiński G . Utility of non-contrast dual-energy CT in thyroid cancer: two cases. Cancer Imaging. (2023) 23:39. doi: 10.1186/s40644-023-00555-w, PMID: 37072868 PMC10114424

[32] Yao C. Chen X. Yang Z. Huang R. Zhang S. Liao Y. . Gemstone Spectral CT Virtual Noncontrast Images and Iodine Maps for the Characterization of Thyroid Lesions and Distinguishing Thyroid Papillary Carcinoma from Nodular Goiter. Int. J. Endocrinol. (2023) 2023:8220034. doi: 10.1155/2023/8220034, PMID: 36891376 PMC9988381

[33] Li F. Huang F. Liu C. Pan D. Tang X. Wen Y. . Parameters of dual-energy CT for the differential diagnosis of thyroid nodules and the indirect prediction of lymph node metastasis in thyroid carcinoma: a retrospective diagnostic study. Gland Surg. (2022) 11(5):913–26. doi: 10.21037/gs-22-262, PMID: 35694089 PMC9177276

[34] Ha T. Kim W. Cha J. Lee Y.H. Seo H.S. Park S.Y. . Differentiating pulmonary metastasis from benign lung nodules in thyroid cancer patients using dual-energy CT parameters. Eur. Radiol. (2022) 32(3):1902–11. doi: 10.1007/s00330-021-08278-x, PMID: 34564746

[35] Zou Y. Sun S. Liu Q. Liu J. Shi Y. Sun F. . A new prediction model for lateral cervical lymph node metastasis in patients with papillary thyroid carcinoma: Based on dual-energy CT. Eur. J. Radiol. (2021) 145:110060. doi: 10.1016/j.ejrad.2021.110060, PMID: 34839216

[36] Yang L. Luo D. Li L. Zhao Y. Lin M. Guo W. . Differentiation of malignant cervical lymphadenopathy by dual-energy CT: a preliminary analysis. Sci. Rep. (2016) 6:31020. doi: 10.1038/srep31020, PMID: 27498560 PMC4976355

[37] Zhao W. Shen S. Ke T. Jiang J. Wang Y. Xie X. . Clinical value of dual-energy CT for predicting occult metastasis in central neck lymph nodes of papillary thyroid carcinoma. Eur. Radiol. (2024) 34(1):16–25. doi: 10.1007/s00330-023-10004-8, PMID: 37526667

[38] Altenbernd J. Wetter A. Umutlu L. Hahn S. Ringelstein A. Forsting M. . Dual-energy computed tomography for evaluation of pulmonary nodules with emphasis on metastatic lesions. Acta Radiol. (2016) 57(4):437–43. doi: 10.1177/0284185115582060, PMID: 25907120

[39] Wu Y.Y. Wei C. Wang C.B. Li N.Y. Zhang P. Dong J.N. . Preoperative Prediction of Cervical Nodal Metastasis in Papillary Thyroid Carcinoma: Value of Quantitative Dual-Energy CT Parameters and Qualitative Morphologic Features. AJR Am. J. Roentgenol. (2021) 216(5):1335–43. doi: 10.2214/AJR.20.23516, PMID: 33760651

[40] He M. Lin C. Yin L. Lin Y. Zhang S. Ma M. . Value of Dual-Energy Computed Tomography for Diagnosing Cervical Lymph Node Metastasis in Patients With Papillary Thyroid Cancer. J. Comput. Assist. Tomogr. (2019) 43(6):970–5. doi: 10.1097/RCT.0000000000000927, PMID: 31738199

[41] Jin D. Ni X. Zhang X. Yin H. Zhang H. Xu L. . Multiphase Dual-Energy Spectral CT-Based Deep Learning Method for the Noninvasive Prediction of Head and Neck Lymph Nodes Metastasis in Patients With Papillary Thyroid Cancer. Front. Oncol. (2022) 12:869895. doi: 10.3389/fonc.2022.869895, PMID: 35515110 PMC9065438

[42] Geng D. Zhou Y. Shang T. Su G.Y. Lin S.S. Si Y. . Effect of Hashimoto’s thyroiditis on the dual-energy CT quantitative parameters and performance in diagnosing metastatic cervical lymph nodes in patients with papillary thyroid cancer. Cancer Imaging. (2024) 24(1):10. doi: 10.1186/s40644-024-00655-1, PMID: 38238870 PMC10797959

